# Supplementary material for: Towards the Standardization of Photothermal Measurements of Iron Oxide Nanoparticles in Two Biological Windows
Source: Nanomaterials (Basel). 2023 Jan 22;13(3):450. doi: 10.3390/nano13030450 (PMC9921180; doi:10.3390/nano13030450)
Supplement: Supplementary file 1 [file nanomaterials-13-00450-s001.zip › nanomaterials-2164783-supplementary.pdf]

SUPPLEMENTARY MATERIAL

Towards the standardization of photothermal measurements of iron oxide nanoparticles in two biological windows.

Daniel Arranz [1,2,3], Rosa Weigand [2] and Patricia de la Presa [1,3,*]

[1] Instituto de Magnetismo Aplicado (UCM-ADIF-CSIC), A6 km. 22.5 Las Rozas (Madrid).; daniarra@ucm.es

[2] Dpto. de Óptica, Facultad de Ciencias Físicas, Universidad Complutense de Madrid, Plaza de las Ciencias 1, 28040 Madrid, Spain; weigand@fis.ucm.es

[3] Dpto. de Física de Materiales, Facultad de Ciencias Físicas, Universidad Complutense de Madrid, Plaza de las Ciencias 1, 28040 Madrid, Spain; pmpresa@fis.ucm.es

* Correspondence: pmpresa@ucm.es;

1. Structural and magnetic characterization

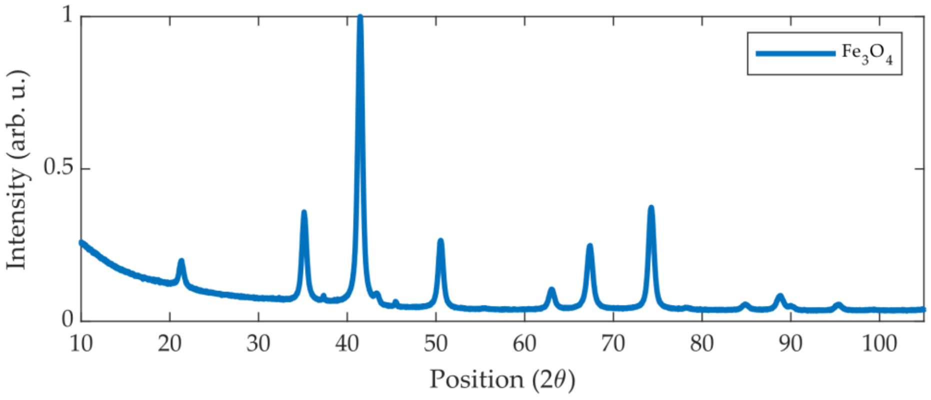

Figure S1. XRD diffractometer pattern of Fe_3O_4.

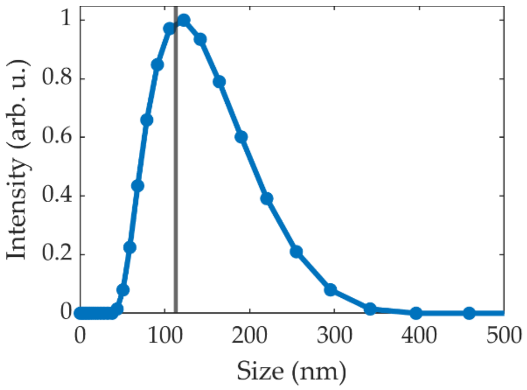

Figure S2. Hydrodynamic size of the colloidal nanoparticle. The black line indicate the mean hydrodynamic size.

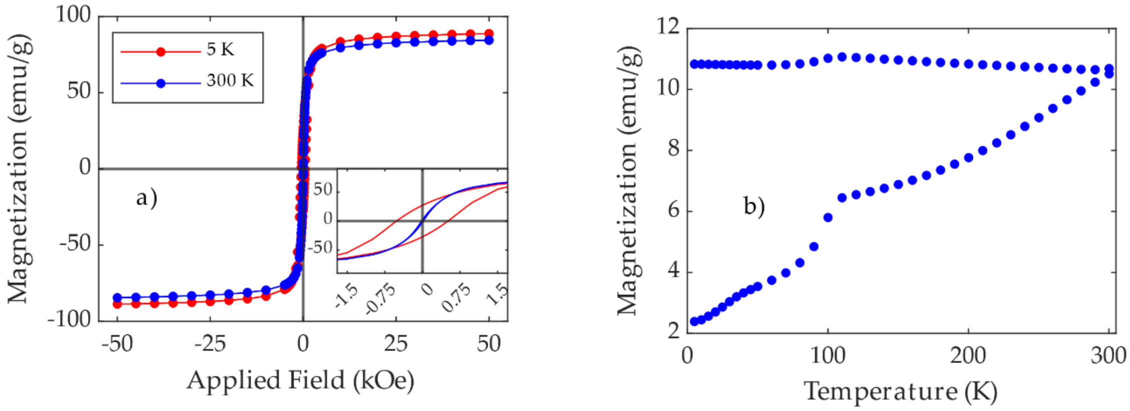

Figure S3. a) Hysteresis loop of Fe_3O_4 at 5 K (red) and 300 K (blue). b) ZFC-FC curves of Fe_3O_4 at 100 Oe.

2. Photothermal characterization

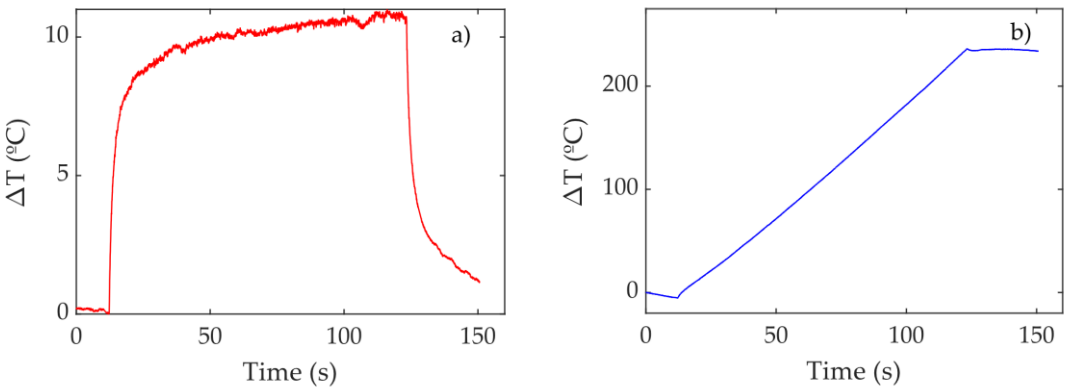

Figure S4. a) Typical thermal curve of a photothermal measure, b) The transformed graph where the linear behavior of ΔT is observed.

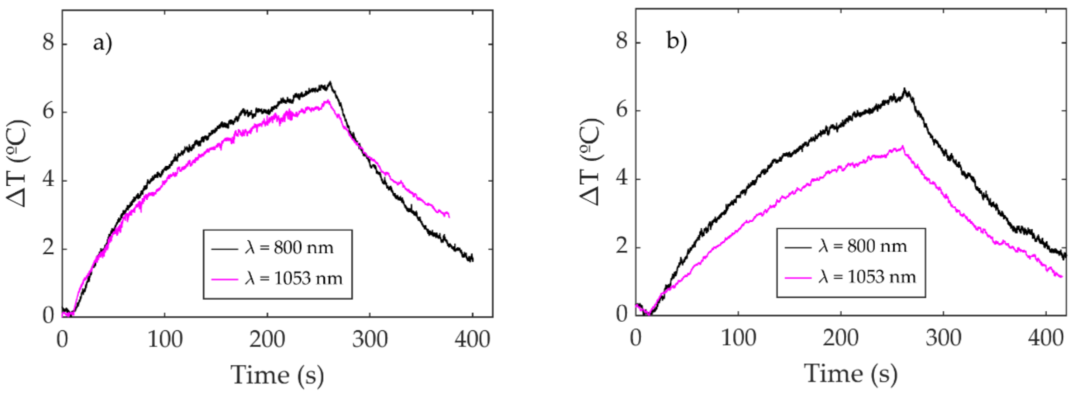

Figure S5. Heating curves for Fe_3O_4 using Eppendorf tubes, irradiated a) from the top and b) from the side. Wavelengths used are 800 nm and 1053 nm, P = 50 mW, spot size S2 and concentration 0.4 mg/mL.

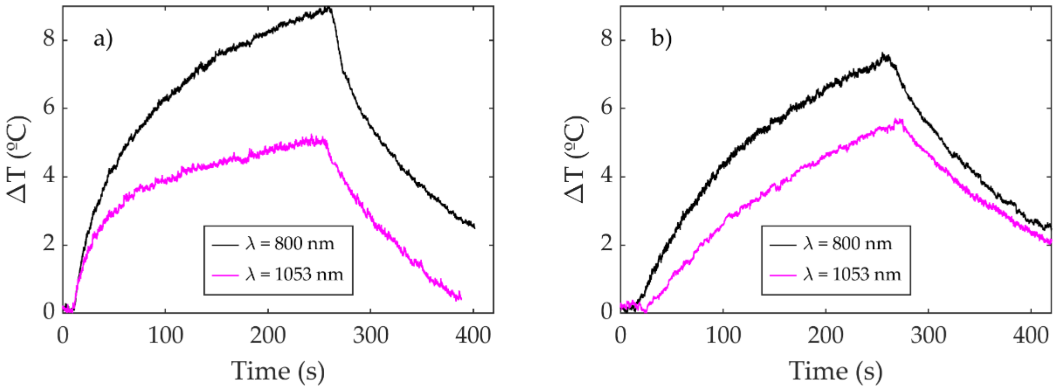

Figure S6. Heating curves for IRA 980B using Eppendorf tubes, irradiated a) from above and b) from the side. Wavelengths used are 800 nm and 1053 nm, P = 50 mW, spot size S2 and concentration 0.4 mg/mL.

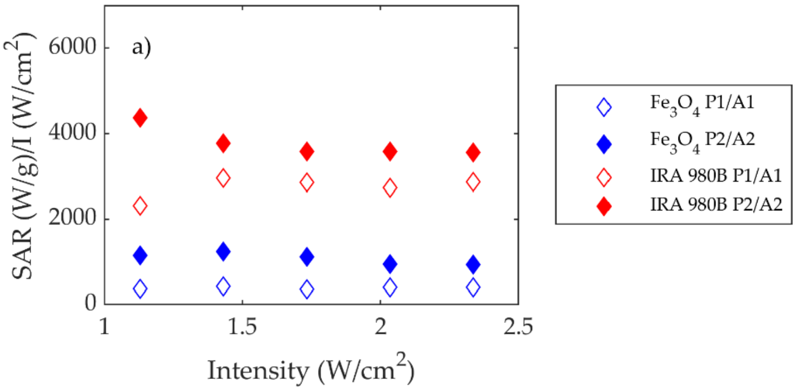

Figure S7. SAR divided by intensity for Fe_3O_4 and IRA 980B using laser at 800 nm and measured in a Teflon vessel.

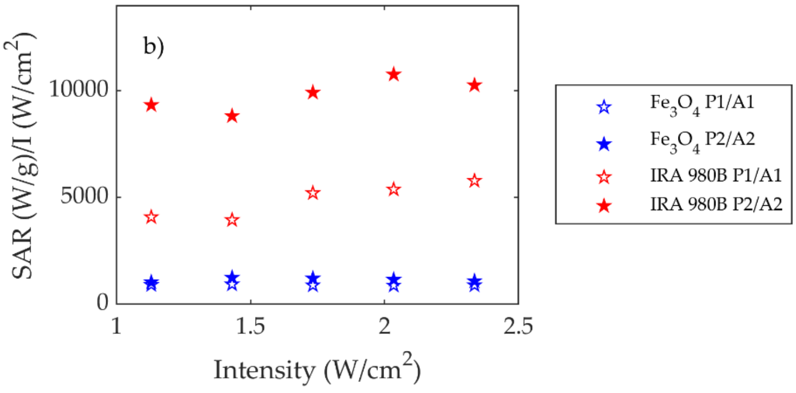

Figure S8. SAR divided by intensity for Fe_3O_4 and IRA 980B using laser at 1053 nm and measured in a Teflon vessel.
